# Beyond conventional drying: improving the quality of dried *Tremella fuciformis* via a heat-integrated pretreatment

**DOI:** 10.1038/s41538-026-00805-8

**Published:** 2026-03-23

**Authors:** Yuanhui Zhang, Nengpai Shi, Cong Yang, Yuankun Jia, Jiaxuan Peng, Xuemei Hou, Shengnan Lin, Xiangyang Lin

**Affiliations:** College of Biological Science and Engineering, Fuzhou University, Fuzhou, Fujian PR China

**Keywords:** Biotechnology, Chemistry, Engineering, Materials science

## Abstract

Hot-air drying (HAD) of *Tremella fuciformis* often results in poor rehydration capacity, yellowing, and long preparation times, which significantly reduce its industrial and consumer value. This study introduces a heat-integrated pretreatment (HIP) as a simple and viable strategy to improve the quality of dried *T. fuciformis*. The effects of HIP on water status, microstructure, and rheological behavior were systematically investigated via using low-field nuclear magnetic resonance (LF-NMR), magnetic resonance imaging (MRI), scanning electron microscopy (SEM), and rheological analysis. HIP effectively regulated water migration and preserved the polysaccharide matrix, which promots a controllable conversion of bound water to free water, thus facilitating more uniform dehydration. Among the tested conditions, HIP at 80 °C for 5 min increased the rehydration capacity by 74% compared with conventional HAD, while maintaining structural integrity and polysaccharide content. The treated samples exhibited brighter appearance, improved gelation behavior, and superior rehydration performance. These findings elucidate the mechanism by which HIP modulates polysaccharide–water interactions during drying and demonstrate its strong potential for manufacturing high-quality, quick-soak dried *T. fuciformis* products with enhanced physicochemical and structural properties.

## Introduction

*Tremella fuciformis* (silver ear fungus) is a jelly-like basidiomycete widely consumed in East and Southeast Asia for both culinary and medicinal purposes^1^. It is traditionally prepared as sweet dessert soups or beverages, where its unique gelatinous mouthfeel is primarily derived from a polysaccharide-rich hydrocolloid matrix. In addition to its sensory appeal, *T. fuciformis* contains diverse bioactive components, including heteropolysaccharides, polyphenols, proteins, and dietary fiber^2,3^. These constituents collectively contribute to a range of health-promoting functions, among which polysaccharides are recognized as the dominant functional components. *T. fuciformis* polysaccharides have been associated with antioxidant^4^, anti-inflammatory^5^, neuroprotective^6^, anti-aging^7^, and gastroprotective activities^8^. Importantly, many of these biological functions are closely linked to the molecular integrity, hydration behavior, and supramolecular organization of the polysaccharide network. These structural features are highly sensitive to thermal and dehydration-induced stresses^9^. Consequently, preservation strategies that maintain polysaccharide structure and water-binding capacity during processing are critical for retaining both functional efficacy and desirable textural properties in dried products.

Fresh *T. fuciformis* contains approximately 90–95% moisture and is highly perishable due to its delicate, gelatinous structure and active postharvest metabolism^10^. If not processed promptly, visible spoilage can occur within 72 h under ambient conditions, primarily driven by enzymatic browning and microbial proliferation^11^. Even under cold storage (4 °C), fresh *T. fuciformis* exhibits a limited shelf life, as browning, odor change, and deterioration can still occur^12^. These rapid physiological changes not only compromise sensory quality but also diminish the nutritional and functional value of the products^11^. Drying is therefore the most widely adopted preservation strategy to enable long-term storage and distribution. However, for hydrocolloid-rich materials such as *T. fuciformis*, inappropriate drying can irreversibly alter water states and induce polysaccharide network collapse. As a result, substantial losses in rehydration performance and functional quality may occur.

Among available techniques, hot-air drying (HAD) remains the dominant industrial method because of its simplicity and low cost^13^. Nevertheless, HAD often induces severe tissue shrinkage, matrix densification, and polysaccharide network collapse, leading to undesirable quality losses such as poor rehydration capacity, texture hardening^12,14,15^, and potential degradation of functional components^16^. Such drawbacks are particularly problematic for instant-prepared products, in which rapid water uptake and recovery of gelatinous texture are key quality attributes. Previous studies have demonstrated that drying behavior and final product quality are influenced not only by drying parameters but also by the pretreatment^17^. Therefore, selecting appropriate pretreatments that stabilize tissue structure and regulate water migration prior to dehydration are essential for improving both processing efficiency and product quality.

Typical heat pretreatment comprises brief thermal exposure in the range of 70–95 °C for less than 10 min. These treatments are widely used in vegetables, fruits, and other plant foods to reduce microbial load and inactivate oxidative enzymes^18^. Such blanching treatments have been shown to extend shelf life in fresh-cut produce, including Chinese cabbage and sweet peppers^19,20^, primarily through the inactivation of polyphenol oxidase and peroxidase. Simultaneously, thermal softening of the cellular matrix enhances permeability and facilitates subsequent dehydration. Importantly, the rate of solute diffusion during thermal exposure was orders of magnitude slower than that of heat transfer, approximately 10³-fold. This disparity allows precise control over enzymatic inactivation while minimizing nutrient leaching^21^. However, these conventional pretreatments are largely designed for enzyme control and microbial safety, rather than for preserving hydrocolloid functionality or regulating polysaccharide-associated water during drying.

To overcome the limitations of conventional drying in preserving the functional and sensory qualities of the dried *T. fuciformis* products, we establish a heat-integrated pretreatment (HIP) strategy for fresh *T. fuciformis*. This approach yields dried products with enhanced rehydration capacity, preserved bioactive substances, and superior structural and sensory attributes. Unlike traditional blanching, which primarily targets enzymatic inactivation, HIP is designed to modulate polysaccharide–water interactions prior to dehydration, by regulating the conversion between bound and free water and stabilizing the polysaccharide network. Through this mechanism, HIP aims to control moisture migration, reduce structural collapse during drying, and ultimately preserve rehydration capacity, bioactive substance retention, and desirable sensory attributes in the dried products. To elucidate the underlying mechanisms, Low-field nuclear magnetic resonance (LF-NMR) and Scanning electron microscopy (SEM) reveal water–biomacromolecule interactions and how HIP maintains tissue integrity, while magnetic resonance imaging (MRI) visualizes spatial moisture migration of fresh *T. fuciformis* during pretreatment. Our findings provide a scalable route to high-quality, quick-soak dried *T. fuciformis* products while deepen the mechanistic understanding of drying-sensitive hydrocolloid-rich foods.

## Results

### HIP for fresh *T. fuciformis*

**Fig. 1:**
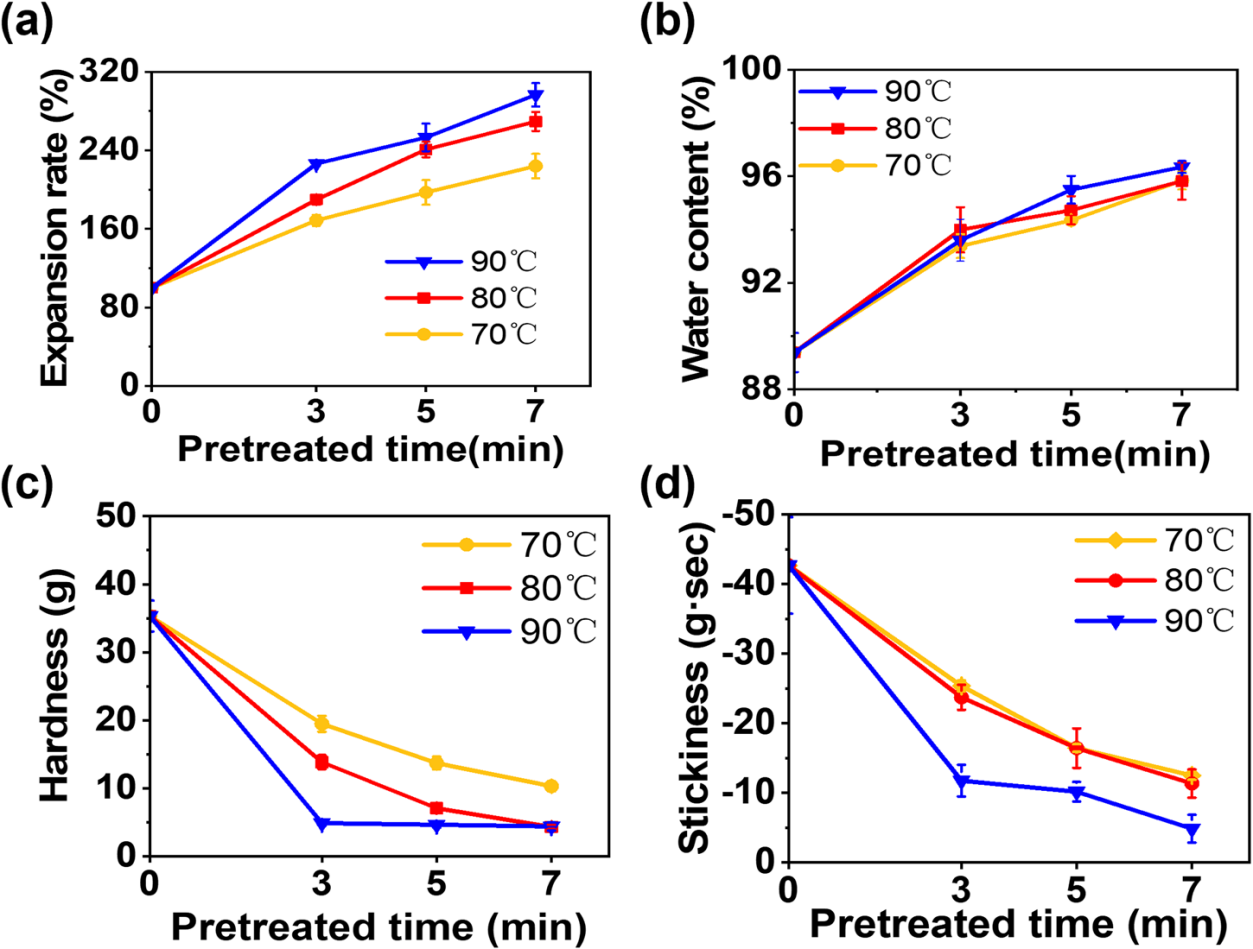
Characteristics of fresh T. fuciformis under different pretreated temperatures and time. **a** Expansion rate, **b** water content, **c** hardness, **d** adhesiveness.

During HIP, heat transfer is accompanied by mass transfer^21^. The pretreated *T. fuciformis* displayed pronounced water absorption and swelling, accompanied by changes in water content, texture, and microstructure. Both pretreatment temperature and time markedly influenced the expansion ratio. As shown in Fig. 1a, with increasing time and temperature, the expansion ratio of fresh *T. fuciformis* significantly induced, reaching a maximum of 297% under 90 °C–7 min conditions. Water content of resulted *T. fuciformis* exhibited a similar trend as shown in Fig. 1b. Meanwhile, at 80 °C for 5 min, the expansion ratio of fresh *T. fuciformis* was 241% and the water content indicated 94.7%.

Thermal processing initiates enzymatic and chemical reactions that alter the quality of plant-derived materials^22,23^. Among these, polysaccharide solubilization and depolymerization are key drivers of the modifications of cell wall and middle lamella, ultimately leading to textural changes^24,25^. As shown in Fig. 1c, d, the HIP markedly reduced both hardness and adhesiveness of the *T. fuciformis* samples, with greater declines observed at higher temperatures and longer duration. At 90 °C, even short treatments presented very low hardness and adhesiveness, while prolonged exposure (7 min) caused destructive damage to the tissue structure. Excessively low hardness and adhesiveness were indicative of overhydration and extensive structural modification, both of which compromised quality and complicated downstream processing. Furthermore, increased water content following severe pretreatment was expected to elevate energy demand during subsequent HAD. Taken together, pretreatment at 80 °C for 5 min achieved an optimal balance, promoting effective expansion while minimizing detrimental textural changes and excessive water uptake, thereby supporting both product quality and processing efficiency.

### Water status and distribution of *T. fuciformis* during pretreatments

**Fig. 2:**
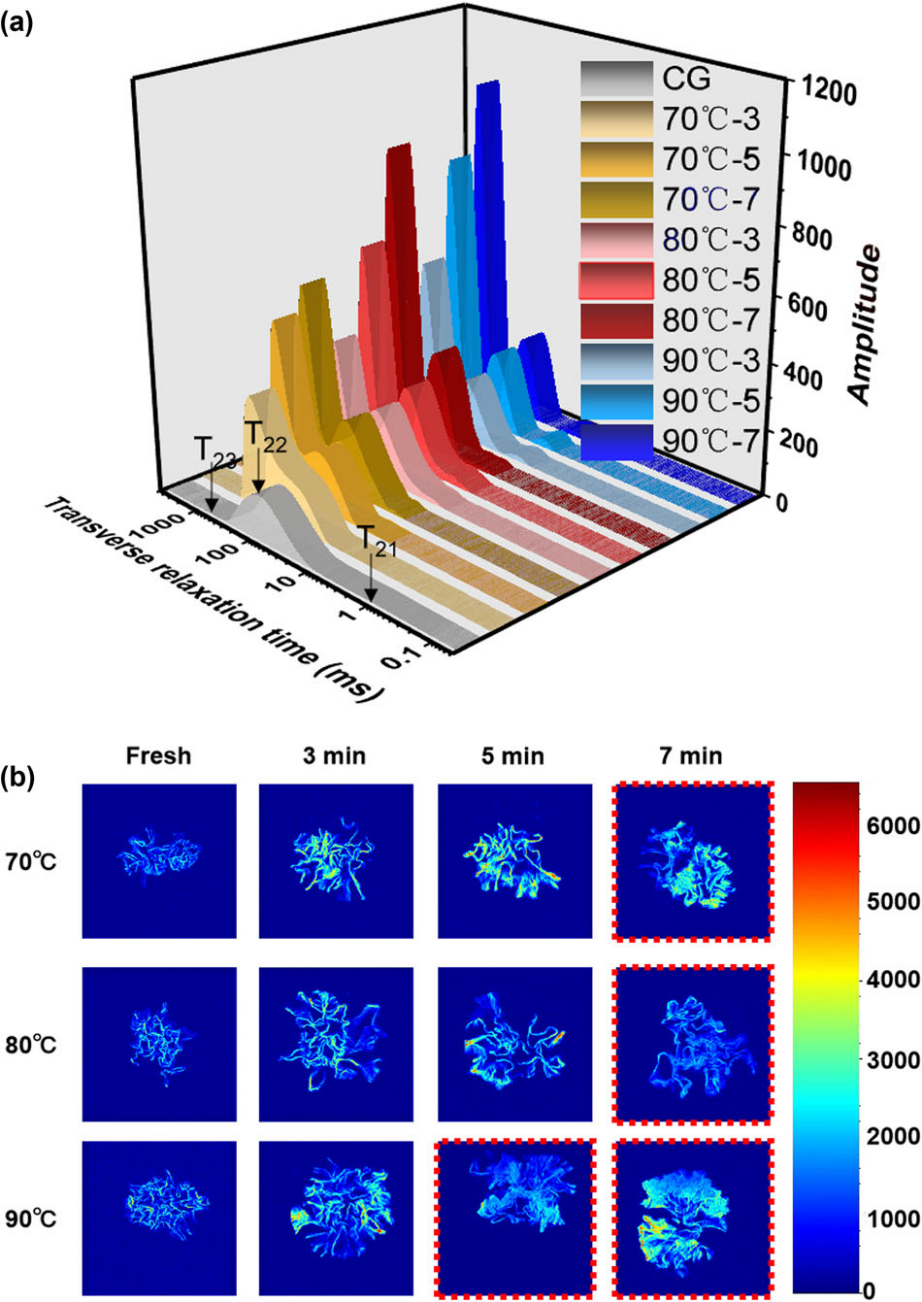
Effects of different heat-integrated pretreatments (HIP) on the Low-field nuclear magnetic resonance (LF-NMR) characteristics of T. fuciformis. **a** Transverse relaxation time (T_2_). **b** The T₂-weighted pseudo-color images of pretreated *T. fuciformis* samples.

LF-NMR has become a powerful non-destructive technique for characterizing water distribution, mobility, and molecular binding states in complex food matrices^26,27^. As shown in Fig. 2a and Supplementary Fig. 1, LF-NMR spectra revealed three discrete water populations in *T. fuciformis*: tightly bound water (T_21_, 0.01–10 ms), weakly bound water (T_22_, 10–100 ms), and free water associated with submicroscopic voids (T_23_, 100–1000 ms)^17^. Shorter T_2_ values indicate stronger molecular binding, while longer T_2_ values denote freer motion of water molecules. In fresh samples, weakly bound water dominated (T_22_), whereas tightly bound (T_21_) and free (T_23_) fractions remained minor, consistent with previous reports^28^. Notably, fresh samples exhibited two small T₂₁ peaks as shown in Supplementary Table 1, corresponding to strong (1.16 ms) and weak (9.72 ms) fractions of tightly bound water^29^. This fraction is mainly associated with hydrogen bonding between water molecules and the polysaccharide-rich cell wall matrix, suggesting the presence of tightly bound water with varying degrees of hydrogen bonding, as the abundant hydroxyl groups of β-glucans and other polysaccharides provide numerous binding sites^30^.

HIP markedly altered water mobility patterns. Both temperature and time significantly influenced relaxation behaviors: T_21_ responded to both factors, T_22_ mainly to temperature, and T_23_ to time as shown in Supplementary Table 1. Across all treatments, rightward shifts in relaxation peaks reflected enhanced proton mobility and weakened molecular interactions. Notably, milder conditions (e.g., 70 °C–3 min) yielded overlapping peaks between bound and free water, suggesting incomplete transition and limited diffusion. Excessive treatments (e.g., 90 °C–7 min) caused pronounced T_23_ expansion and signal broadening, indicative of structural collapse and uncontrolled hydration due to network degradation. 80 °C–5 min treatment produced moderate T_2_ elongation with a balanced redistribution from M_22_ (weakly bound) to M_23_ (free water), signifying controlled water release without over-hydration. This equilibrium favored effective mass transfer and drying kinetics.

MRI is a non-destructive technique widely used to visualize spatial water distribution and mobility in food tissues^31^. In proton density-weighted images, signal intensity is closely related to local water content and molecular mobility^32^. As shown in Fig. 2b, the 80 °C–5 min samples exhibited uniform brightness distribution, reflecting homogeneous water mobility and intact cellular architecture. In contrast, prolonged heating led to heterogeneous intensities and darkened regions, denoting localized collapse and abnormal water accumulation.

Together, the LF-NMR and MRI analyses confirm that moderate HIP, specifically, the 80 °C–5 min samples, optimizes water redistribution by converting a portion of bound water into mobile fractions. This controlled migration underpins the efficient drying and desirable texture for producing high-quality dried *T. fuciformis*.

### Mechanisms of HIP for processing fresh *T. fuciformis*

**Fig. 3:**
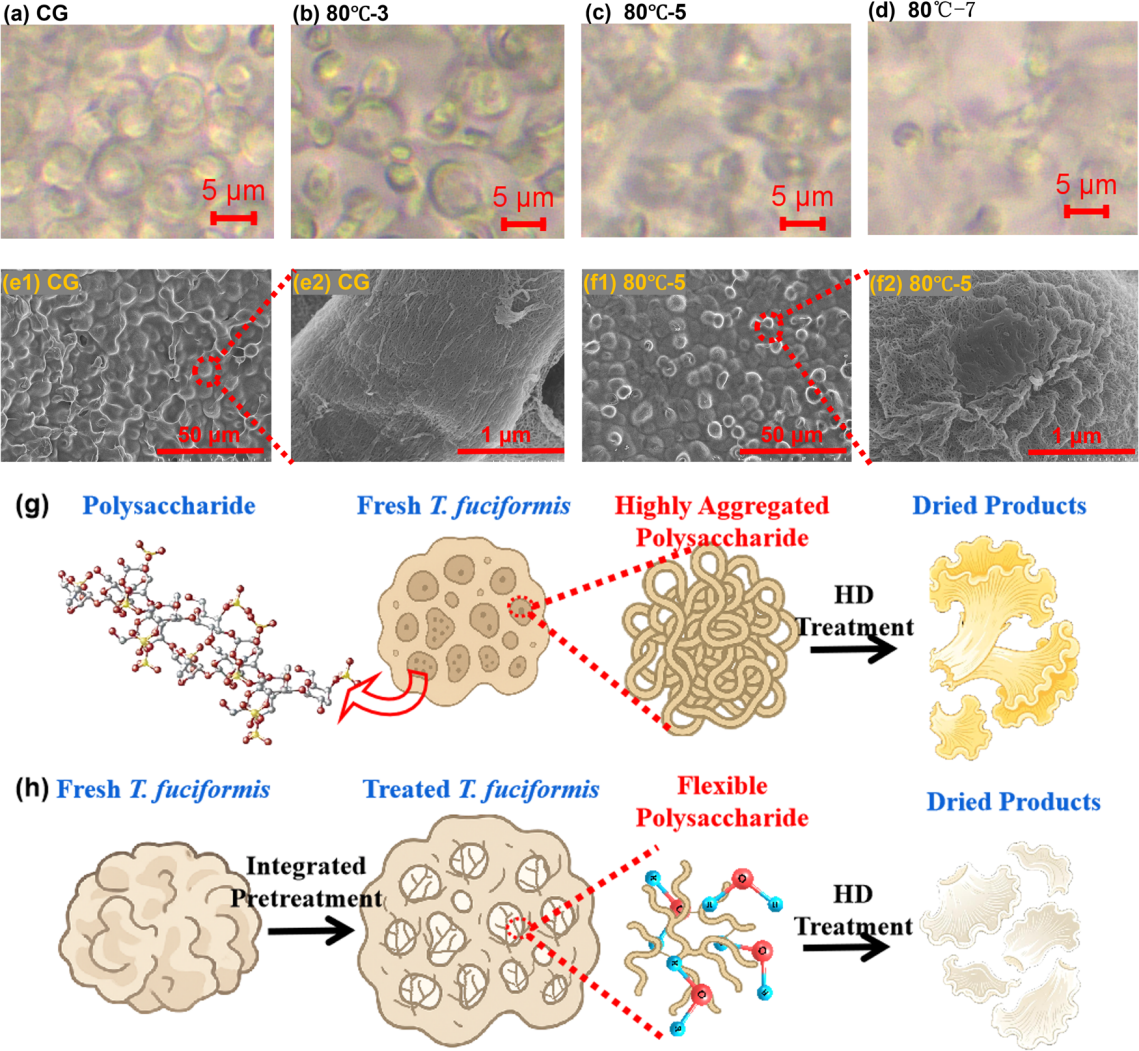
Proposed mechanisms for processing high-quality T. fuciformis products. **a**–**d** Optical microscopy results of control group (CG) vs. different pretreated *T. fuciformis* samples under 80 °C, **e**1, **e**2, **f**1, **f**2 SEM images of CG vs. pretreated *T. fuciformis* samples at 80 °C–5 min condition. Schematic illustration fresh *T. fuciformis* of **g** under conventional drying processing or **h** with suitable HIP.

As shown in Fig. 3a–d, optical microscopy images revealed distinct microstructural changes in *T. fuciformis* subjected to HIP at 80 °C compared with the CG. HIP induced progressive loosening of the polysaccharide network, whereas the untreated sample (CG) maintained a compact cellular structure. The optimal effect was achieved with a 5-min pretreatment at 80 °C, which caused pronounced tissue expansion and enhanced porosity. In contrast, prolonged and high temperature pretreatment (80 °C–7 min) caused evident structural collapse and aggregation, indicating over-disruption of the native gel-like framework.

As shown in Fig. 3e1-e2, f1-f2, SEM observations provided further insight into these changes. The CG sample exhibited tightly packed, aggregated polysaccharide fibrils with limited void space, which were unfavorable for water mobility and mass transfer during drying. By comparison, the 80 °C–5 min treated sample showed a looser and more porous architecture, facilitating water redistribution and diffusion. These findings aligned with the LF-NMR and MRI results (Fig. 2), confirming that appropriate pretreatment promotes bound-to-free water transformation while preserving structural integrity.

Collective evidence from LF-NMR, MRI, and SEM analyses supports the proposed mechanism by which HIP maintained the structural integrity of *T. fuciformis*. As shown in Fig. 3g, h, polysaccharides, the predominant solids in *T. fuciformis*, form a hydrophilic network that governs water binding and mobility during drying, due to their strong interaction with water molecules and effect on water distribution^9,33^. In the conventional drying pathway, polysaccharides in the fresh *T. fuciformis* existed in a highly aggregated state, restricting water mobility and leading to uneven dehydration, textural hardening, and reduced rehydration capacity of the dried products. In contrast, under optimized HIP conditions, the polysaccharide chains became more flexible and spatially expanded, increasing available binding sites and enhancing water migration during drying. These results highlight that moderate HIP effectively balances water migration and structural stability, whereas excessive pretreatment disrupts the polysaccharide–water molecules system and compromises product quality.

### Drying characteristics of different pretreated *T. fuciformis*

**Fig. 4:**
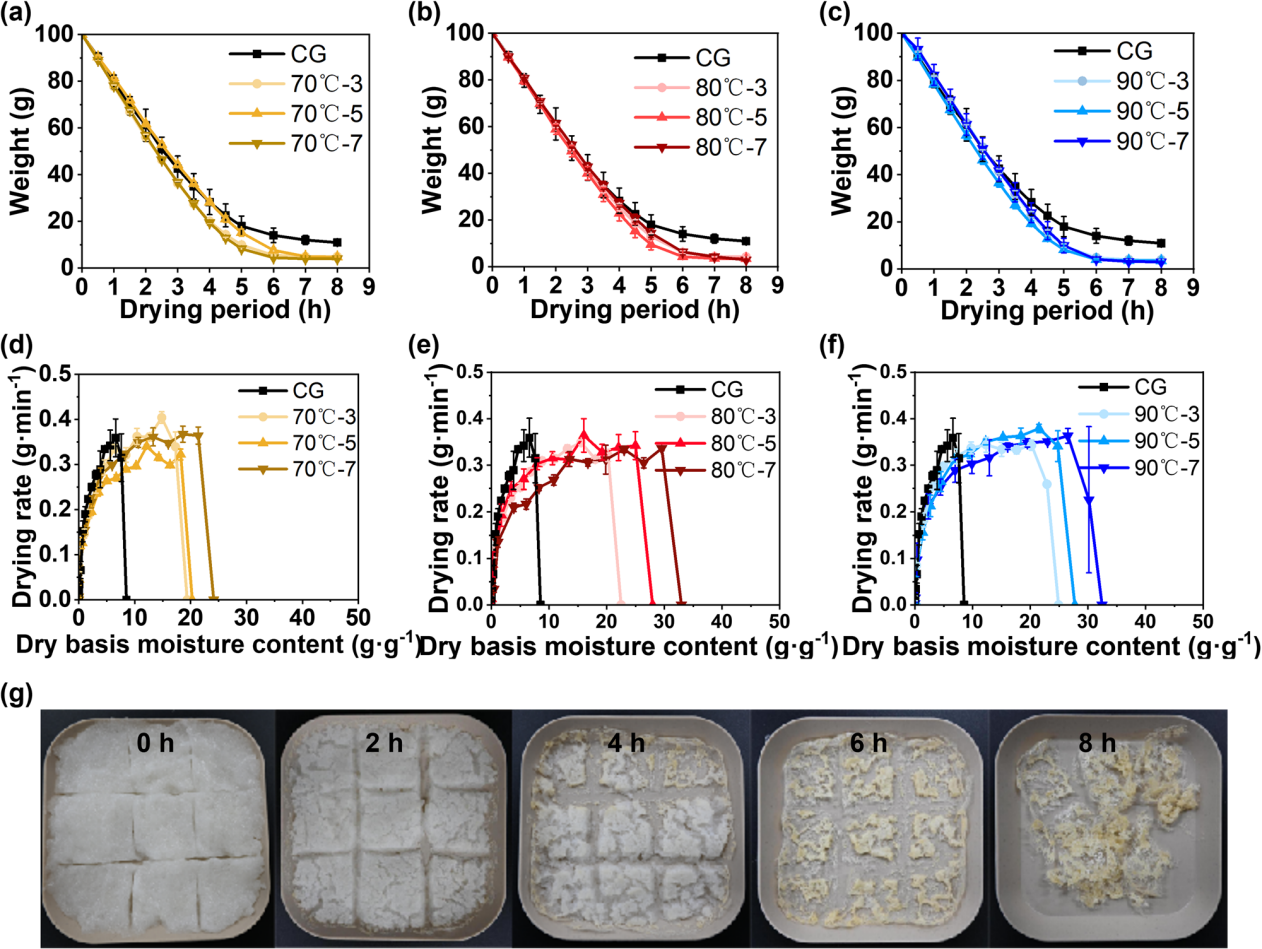
Drying behavior of T. fuciformis under heat-integrated pretreatments (HIP). **a**–**c** Drying curves and **d**–**f** drying rates of *T. fuciformis* under HIP with different temperatures and times. **g** Drying processing of *T. fuciformis* pretreatment at 80 °C for 5 min, photographed at 2 h intervals.

The quality of the final product and the drying rate are determined not only by drying conditions but also by pretreatment parameters such as temperature and time^34^. The drying behavior of *T. fuciformis* was strongly affected by pretreatment conditions. As shown in Fig. 4 and Table 1, The pretreated *T. fuciformis* samples consistently exhibited higher dehydration rates than the control group during subsequent HAD (at 75 °C for 8 h). Under identical drying conditions, all samples reached constant weight at 8 h, however, the drying kinetics differed. These results indicate that HIP enhances dehydration efficiency within a suitable temperature–time window, consistent with previous reports on blanched red bell pepper^35^.

**Table 1:** Nonlinear fitting results of hot-air drying (HAD) of *T. fuciformis* under different heat-integrated pretreatment (HIP) conditions

Model	Groups		Model parameters	R²	RMSE	χ²
	Temperature	Time				
Page (MR=exp.(-kt^n^)	CG		*k* = 0.229, *n* = 1.412	0.998	0.016	3.15E-03
	70 °C	3 min	*k* = 0.228, *n* = 1.494	0.996	0.022	5.58E-03
		5 min	*k* = 0.181, *n* = 1.501	0.995	0.025	7.44E-03
		7 min	*k* = 0.217, *n* = 1.524	0.995	0.025	7.65E-03
	80 °C	3 min	*k* = 0.190, *n* = 1.514	0.996	0.023	6.45E-03
		5 min	*k* = 0.194, *n* = 1.536	0.995	0.027	8.59E-03
		7 min	*k* = 0.187, *n* = 1.470	0.996	0.024	6.67E-03
	90 °C	3 min	*k* = 0.165, *n* = 1.652	0.996	0.024	7.03E-03
		5 min	*k* = 0.214, *n* = 1.531	0.996	0.023	6.43E-03
		7 min	*k* = 0.165, *n* = 1.628	0.996	0.022	5.93E-03
Logarithmic (MR = a exp.(-kt) + c	CG		*k* = 0.302, *a* = 1.176, *c* = −0.125	0.991	0.036	1.53E-02
	70 °C	3 min	*k* = 0.297, *a* = 1.230, *c* = −0.177	0.988	0.042	1.91E-02
		5 min	*k* = 0.216, *a* = 1.339, *c* = −0.297	0.991	0.036	1.39E-02
		7 min	*k* = 0.288, *a* = 1.244, *c* = −0.192	0.986	0.045	2.27E-02
	80 °C	3 min	*k* = 0.238, *a* = 1.305, *c* = −0.258	0.990	0.039	1.63E-02
		5 min	*k* = 0.252, *a* = 1.288, *c* = −0.239	0.987	0.044	2.13E-02
		7 min	*k* = 0.217, *a* = 1.324, *c* = −0.286	0.992	0.033	1.19E-02
	90 °C	3 min	*k* = 0.242, *a* = 1.333, *c* = −0.268	0.982	0.052	2.92E-02
		5 min	*k* = 0.288, *a* = 1.246, *c* = −0.191	0.986	0.045	2.23E-02
		7 min	*k* = 0.236, *a* = 1.341, *c* = −0.274	0.985	0.048	2.52E-02

Further analysis was conducted based on the dehydration rate curves. Increasing blanching temperature and duration led to a simultaneous increase in the initial moisture content of the samples. Nevertheless, the HAD process generally followed the typical drying kinetics observed in conventional dehydration, which can be divided into three stages: an accelerating period, a constant-rate period, and a falling-rate period as shown in Fig. 4d–f. The accelerating stage was extremely short, and no significant differences were observed among treatments during the falling-rate stage. During the constant-rate drying phase, the 70 °C–5 min group exhibited a relatively lower moisture loss rate compared with other samples within the same set of curves, whereas the 80 °C–5 min and 90 °C–5 min groups showed higher dehydration rates. In addition, Fig. 4g demonstrates that throughout the drying process, the surface of the *T. fuciformis* slurry gradually lost moisture, resulting in cracking and volume shrinkage. Meanwhile, slight yellow–brown discoloration developed progressively from the edges.

During HAD, pretreated *T. fuciformis* samples exhibited markedly higher initial moisture contents than the untreated control, owing to water absorption and swelling during HIP. As a result, the drying curves shifted rightward, and the constant-rate phase was substantially prolonged as shown in Fig. 4d–f. Overall, HIP increased the initial water loading but simultaneously altered the internal mass-transfer behavior, with the extent of these effects depending strongly on temperature–time combinations.

Among all treatments, 80 °C–5 min treatment resulted in more favorable drying kinetics. Compared with the control, this condition accelerated the drying rate while largely preserving the macroscopic structure of dried *T. fuciformis*. As shown in Fig. 4g and Supplementary Fig. 2, 80 °C–5 min samples exhibited moderate and uniform volume shrinkage, regular cracking, and minimal browning even after prolonged drying (6 h). These observations indicate that the 80 °C–5 min treatment achieves an optimal balance between enhanced dehydration efficiency and structural stability.

As shown in Table 1, to quantitatively evaluate the effects of HIP on the drying kinetics of *T. fuciformis*, both the Page and logarithmic models were applied for nonlinear fitting of the HAD data. The Page model exhibited superior fitting performance across all treatments, with determination coefficients (R²) ranging from 0.995 to 0.998, root mean square errors (RMSE) between 0.016 and 0.027, and chi-square (χ²) values as low as 3.15–8.59 (*10^3^), indicating markedly better predictive capability than the logarithmic model. Within the Page model framework, *k* represents the exponent of the drying rate, *n* is an empirical exponent describing the curvature of the drying curve and the degree of deviation from simple exponential drying, often associated with diffusion-controlled moisture transport during the falling-rate period^36^. *n* > 1.0 reflects a typical falling-rate drying behavior. *n* of pretreated samples were consistently higher than that of the CG (*n* = 1.412), The elevated *n* values further indicate an enhanced contribution of internal moisture diffusion to the overall drying process^36^, which can be attributed to structural relaxation of the cell wall matrix. This relaxation facilitates the release of free water, reduces internal mass transfer resistance, and improves surface evaporation efficiency. Among all conditions, the 80 °C–5 min pretreatment demonstrated more balanced and stable kinetic performance. Although its drying rate constant (*k* = 0.194) was slightly lower than that of certain short-time high-temperature groups, its combination of higher *n* (1.536), excellent R² (0.995), low RMSE (0.027), and small χ² (8.59 × 10^−3^) indicates that the drying process under this condition is both efficient and kinetically stable.

Overall, these findings confirm that HIP enhances dehydration by modifying water-binding states and improving diffusivity. The 80 °C–5 min condition achieves an optimal kinetic balance—maintaining high drying efficiency while preserving the structural integrity essential for product quality.

### Comprehensive quality characterization of *T. fuciformis* following HIP

To comprehensively evaluate the effects of HIP on the overall quality of *T. fuciformis*, multiple analytical approaches were employed, including colorimetric analysis, rehydration performance, polysaccharide content, and rheological assessment of the reconstituted soups. These complementary characterizations collectively elucidate how HIP modulates product appearance, functional component retention, and structural functionality, thereby determining consumer-relevant quality attributes.

### Color properties

Color is an important indicator of product quality and a critical determinant of consumer acceptance. In this study, the color parameters (L*, a*, b*), chroma (C), and total color difference (ΔE) of *T. fuciformis* are evaluated after different pretreatments, with the untreated control group (CG) used as reference.

As shown in Table 2, HIP significantly altered the visual characteristics of *T. fuciformis*, showing a distinct intensity-dependent trend: moderate treatments increased brightness, resulting in a more desirable appearance. Specifically, all HIP conditions markedly increased L* values, with the 90 °C–5 min group showing the most pronounced improvement (L* = 64.66), a 20.86% increase over the control. This brightening effect can be attributed to enhanced translucency due to water absorption during blanching. Increase in brightness was generally accompanied by reduced color saturation. Chroma values declined significantly across all treatments and decreased progressively with higher temperature and longer duration. The 90 °C–7 min group exhibited the lowest chroma (*C* = 6.86), a 57.78% reduction, it may be due to pigment degradation caused by thermal effects and the collapse of the tissue structure.

**Table 2:** Color changes of dried *T. fuciformis* via different pretreatments

Groups		L*	a*	b*	ΔE	C	Appearance
Temperature	Time						
CG		53.50 ± 1.40	4.28 ± 0.27	15.68 ± 0.92	-	16.25 ± 0.91	
70 °C	3 min	58.12 ± 1.11b	1.41 ± 0.28a	12.22 ± 1.40a	6.94 ± 0.35b	12.30 ± 1.42a	
	5 min	55.38 ± 2.72c	0.97 ± 0.16b	9.62 ± 1.29b	6.48 ± 1.13b	10.33 ± 0.94b	
	7 min	62.47 ± 1.97a	0.96 ± 0.41b	12.13 ± 1.41a	11.06 ± 1.77a	11.51 ± 0.48a	
80 °C	3 min	52.60 ± 1.21c	1.51 ± 0.07a	10.67 ± 0.76a	4.67 ± 0.64c	10.77 ± 0.75a	
	5 min	57.72 ± 2.01b	0.99 ± 0.10b	9.06 ± 1.04b	8.44 ± 0.86b	9.12 ± 1.02b	
	7 min	61.40 ± 3.08a	0.07 ± 0.19c	7.39 ± 0.79c	12.41 ± 1.77a	7.40 ± 0.79c	
90 °C	3 min	57.54 ± 0.30c	0.77 ± 0.03a	9.07 ± 0.51a	8.16 ± 0.53c	9.10 ± 0.51a	
	5 min	64.66 ± 1.78a	−0.04 ± 0.04c	8.55 ± 0.97a	14.36 ± 1.12a	8.55 ± 0.91a	
	7 min	61.76 ± 2.56b	0.01 ± 0.03b	6.86 ± 1.89b	13.08 ± 0.82b	6.86 ± 1.89b	

Data are mean ± SD (*n* = 9), analyzed by t-test. Different letters within a column indicate significant differences at the same temperature but different times (*p* < 0.05).

The total color difference (ΔE) further confirmed this trade-off, increasing with pretreatment intensity. The largest deviations were found in the 90 °C–5 min (ΔE = 14.36) and 90 °C–7 min (ΔE = 13.08) groups, indicating that high-intensity blanching produced excessive color distortion. In contrast, the 80 °C–5 min pretreatment achieved the best balance between improved lightness and acceptable chroma retention. These results demonstrate that moderate HIP effectively suppresses enzymatic browning and enhances brightness without inducing color fading or visual deterioration.

### Rehydration behavior and polysaccharide retention

**Fig. 5:**
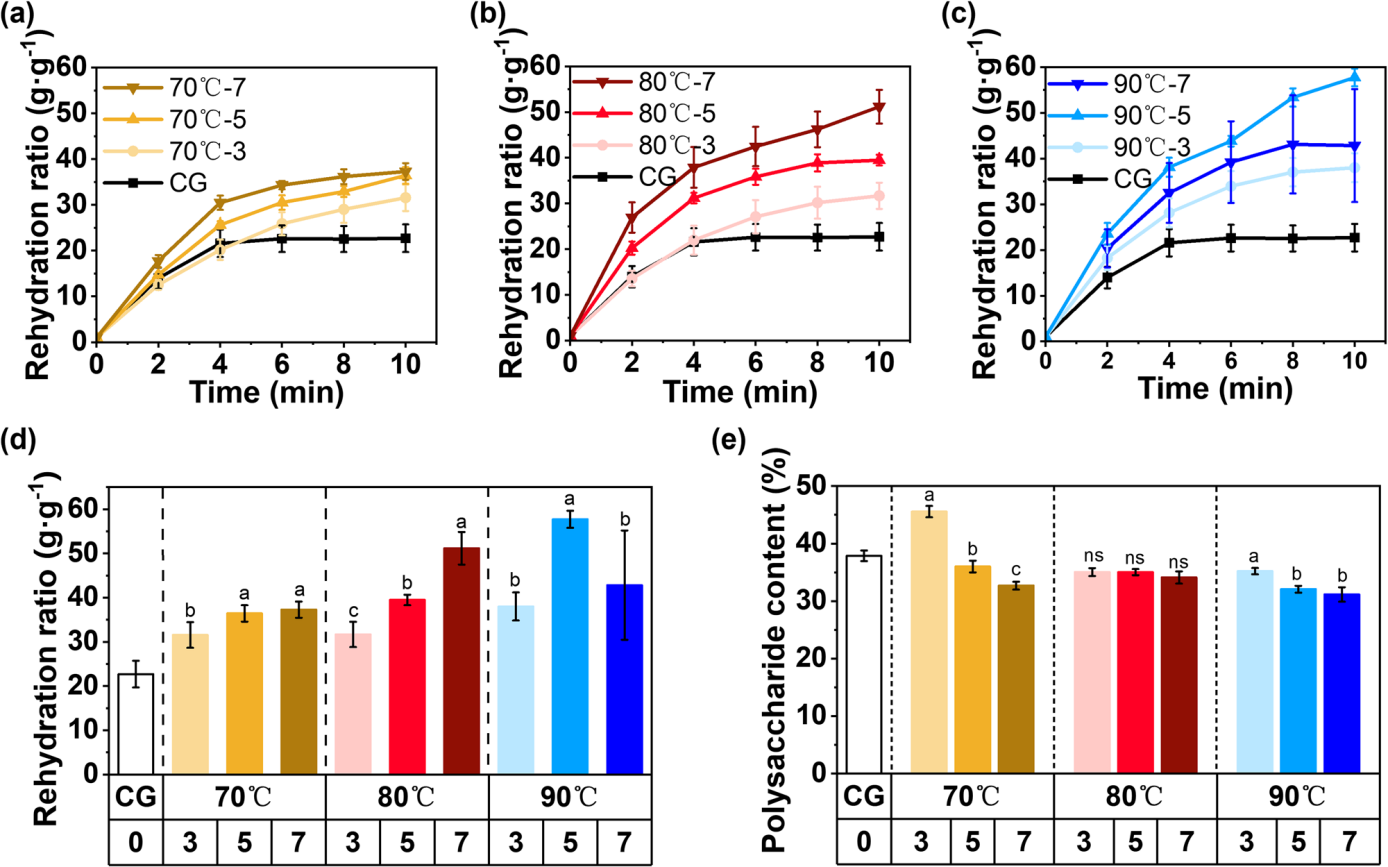
Rehydration characteristics and polysaccharide content of dried T. fuciformis products. **a**–**c** Rehydration curves of different dried *T. fuciformis* products. **d** Comparison of their rehydration ratio at 10 min. **e** Influence of pretreatments on polysaccharide content of the dried *T. fuciformis* products.

Rehydration capacity is a critical quality indicator for dried *T. fuciformis*, as it directly reflects the structural integrity and water-holding ability of the polysaccharide-rich matrix^37^. As shown in Fig. 5a–c, all HIP-pretreated samples presented markedly higher rehydration ratios compared with the untreated control, demonstrating that HIP facilitated water absorption during rehydration. The rehydration process followed a rapid increase within the first 6 min, followed by a gradual plateau phase, which is consistent with typical diffusion-driven hydration behavior observed in porous plant- and fungal-derived materials^38^.

As shown in Fig. 5d, the quantitative comparison at 10 min further revealed that the enhancement in rehydration capacity was strongly dependent on pretreatment temperature and duration. The 90 °C–5 min group achieved the highest rehydration ratio (57.7 g g^−1^), which was 154% higher than that of the control group (22.7 g g^−1^) (calculated as the percentage increase of the treated group’s rehydration ratio relative to the control), while both shorter (3 min) and longer (7 min) pretreatments at the same temperature yielded slightly lower values. This indicates that moderate pretreatment conditions (80 °C–5 min) promote optimal loosening of the fungal microstructure, thereby enhancing water diffusion pathways, whereas excessive heating such as 90  °C treatment may cause structural collapse and reduced rehydration efficiency.

As shown in Fig. 5e, Polysaccharide content showed a decreasing trend with increasing pretreatment severity, reflecting partial leaching of soluble components during pretreatment. Nevertheless, moderate pretreatment (80 °C–5 min) maintained relatively high polysaccharide retention while delivering superior rehydration performance. This balance suggests that excessive pretreatment sacrifices bioactive polysaccharides without proportionally improving rehydration. The optimal conditions (80 °C–5 min) allow preservation of functional components while enhancing structural rehydration ability.

As shown in Supplementary Fig. 3, the water activity of dried *T. fuciformis* further illustrates the stabilizing role of HIP. After two and eight weeks of sealed refrigerated storage, HIP-treated samples consistently exhibited lower water activity than the untreated control, reflecting stronger water binding and more uniform dehydration. Moderate pretreatment presented the lowest and most stable values (<0.45), whereas excessive treatment slightly increased water activity due to partial polysaccharide degradation and exposure of hydrophilic sites. Over time, all samples reached equilibrium, with the control stabilizing at 0.561 and treated samples remaining below the microbial safety threshold of 0.6^39^. These results demonstrate that the optimized HIP not only enhances dehydration and polysaccharide preservation but also ensures long-term physicochemical of dried *T. fuciformis*. and remained below the microbial safety threshold of water activity 0.6, suggesting potential microbial stability.

### Rheological behavior of reconstituted *T. fuciformis* soups

**Fig. 6:**
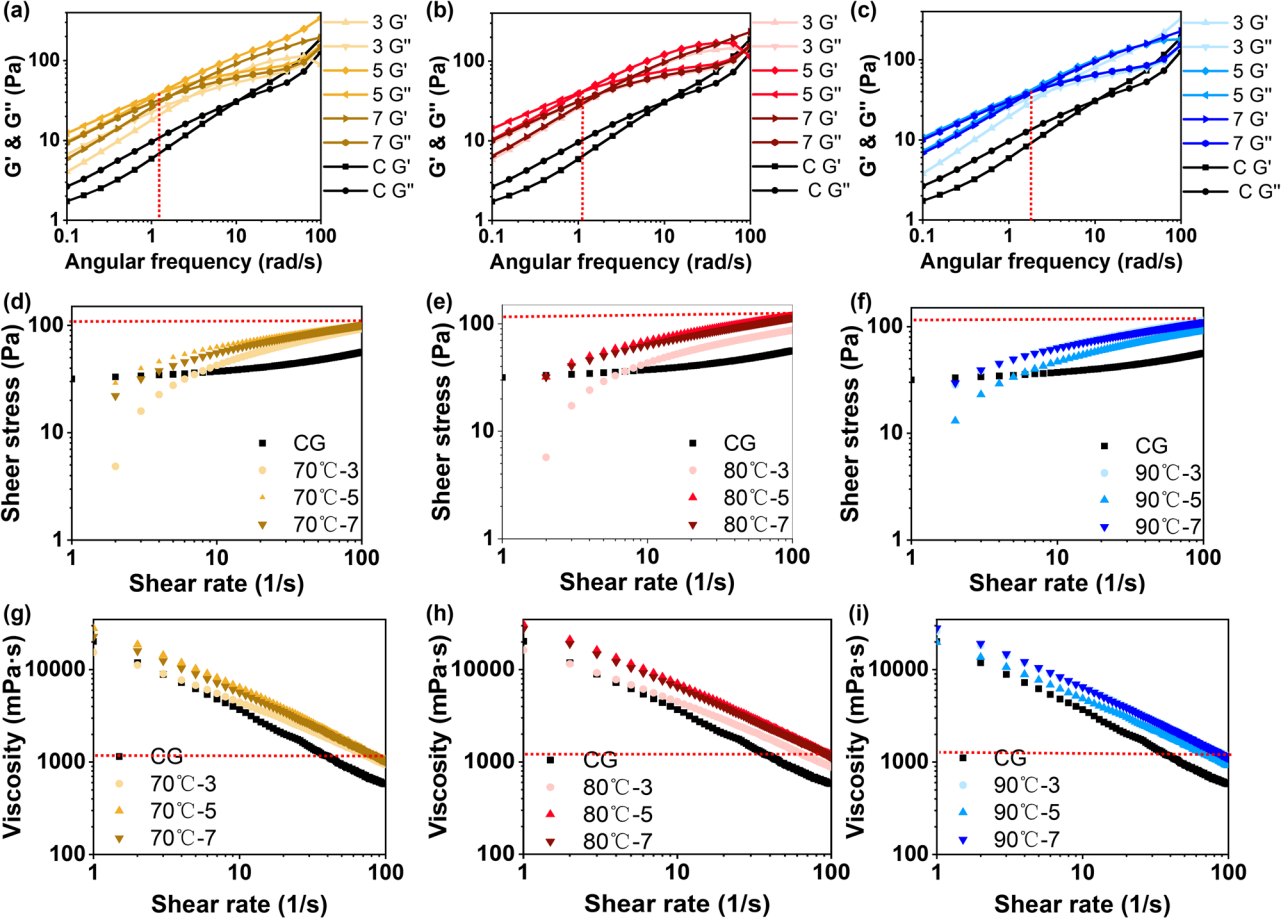
Rheological properties of instant-prepared T. fuciformis soup from dried samples. **a**–**c** Dynamic rheological curves, **d**–**f** shear stress–shear rate curves, and **g**–**i** apparent viscosity–shear rate curves of different soups prepared under different pretreatment conditions.

The rheological behavior of instant-prepared *T. fuciformis* soups provides important insight into the molecular structure and interaction state of polysaccharides released during reconstitution. As shown in Fig. 6a–c, both the storage modulus (G′) and loss modulus (G″) increased progressively with angular frequency, indicating a typical frequency-dependent viscoelastic response characteristic of weak gel-like polysaccharide systems. This is consistent with previous studies^40^, where the transition from low-frequency samples to high-frequency gels is related to the properties of polysaccharides. The control group exhibited the highest crossover frequency (10.76), suggesting that viscous behavior predominated and that the polysaccharide network formed during rehydration was relatively weak. In contrast, HIP-treated samples showed significantly lower crossover frequencies (1.11–2.95), reflecting a pronounced shift toward elastic-dominant behavior and improved gel-forming capacity. Among all treatments, 80 °C–5 min yielded the lowest crossover frequency (1.11), implying the establishment of stronger transient polysaccharide networks with enhanced structural stability. This result highlights that moderate HIP can effectively promote intermolecular associations and reinforce the viscoelastic framework of the reconstituted soup.

As shown in Fig. 6d–i, steady shear tests further demonstrated that all samples exhibited pronounced shear-thinning behavior, which is a hallmark of polysaccharide-rich dispersions and reflects the progressive alignment and disentanglement of polymer chains under increasing shear. Compared with the control, HIP-treated soups showed higher apparent viscosity and shear stress, confirming strengthened intermolecular interactions and greater resistance to flow. Notably, the 80 °C–5 min and 70 °C–5 min groups achieved the highest apparent viscosity. According to Ma et al.’s research^41^, linear molecules require more rotational space than highly branched molecules, thus, solutions with a highly branched structure typically have lower viscosity than those with linear molecules of the same molecular weight. Therefore, dried tremella with HIP may release more linear polysaccharide chains after preparing as a soup.

These rheological trends are consistent with the polysaccharide retention results. Moderate HIP conditions preserved the polysaccharide matrix and promoted functional network formation, while excessive pretreatment led to degradation and viscosity loss. Collectively, the enhanced viscoelasticity and shear stability observed under optimized HIP conditions may contribute to improved textural attributes and consumer-perceived mouthfeel of instant *T. fuciformis* soup products.

## Discussion

HIP represents a mechanistically informed approach that goes beyond conventional HAD by actively regulating water mobility, structural evolution, and polysaccharide functionality in *T. fuciformis*. In this study, HIP at 80 °C for 5 min was identified as the optimal condition, the primary mechanism involved the regulation of polysaccharide–water interactions, which promoted network expansion and increased tissue porosity, thereby facilitating the conversion of bound water into more mobile free water. This shift accelerated dehydration efficiency while maintaining cellular integrity. As a result, the optimized HIP treatment improved rehydration capacity, preserved polysaccharide retention, and enhanced rheological performance of instant-prepared soups, indicating more desirable textural stability. In contrast, excessive thermal exposure disrupted microstructural organization, intensified polysaccharide degradation, and reduced functional performance, highlighting the importance of balancing pretreatment intensity to avoid compromising quality. Overall, these findings demonstrate that HIP provides a scalable and physicochemically guided approach for improving the structural and functional potential of mushroom-derived foods. HIP offers a promising strategy to achieve both processing efficiency and high-value product quality beyond conventional drying practices. Future work should further evaluate sensory attributes and bioactivity changes to strengthen the broader food chemistry significance of HIP-treated products.

## Methods

### Experimental materials

Fresh *Tremella fuciformis* (silver ear fungus) (provided by Fujian Sheng’er Biotechnology Co., Ltd.) with intact fruiting bodies and uniform size (13–17 cm) and smooth surfaces, stored under refrigeration and experimented within 72 h of harvesting. Phenol (CAS: 108-95-2) was provided by Macklin Biochemical Co., Ltd., Shanghai, China. Sulfuric acid 98% (CAS: 7664-93-9) was purchased from Sinopharm Chemical Reagent Co., Ltd., Shanghai, China. Formaldehyde solution 4%, buffered, Ph 6.9, was purchased from Biosharp Biochemical Co., Ltd., Shanghai, China. All of the other regents were of analytical grade or above.

### HIP and drying for *T. fuciformis*

According to previous reports^34^, the intact fresh *T. fuciformis* samples were blanched in a water bath at different temperatures (70 °C, 80 °C, 90 °C) for various durations (3 min, 5 min, 7 min). After blanching, the samples were immediately removed, drained, and analyzed for water absorption expansion ratio and moisture content^28^. To simulate instant *T. fuciformis* instant soup products. The samples were then homogenized into a slurry, evenly spread on food-grade polypropylene trays (13 × 13 cm, ca. 1 cm thickness, 100 ± 0.2 g per tray), and subjected to HAD (DHG-9070, Yiheng Scientific Instruments Co., Ltd., Shanghai, China) at 75 °C.

During the initial drying stage, samples were weighed every 30 min; after 5 h, weighing intervals were extended to 1 h until achieving near-constant weight. During drying period, the samples were flipped to ensure uniform drying.

### Drying characteristics

Drying curves were plotted based on weight changes. The dry-basis moisture content and dehydration rate during HAD were calculated by using Eqs. (1) and (2), respectively^40^.

1 $$\omega =\frac{{m}_{t}-{m}_{g}}{{m}_{g}}$$

Where ${\rm{\omega }}$ indicates dry-basis moisture content(g g^−1^), m_g_ defines as mass of absolutely dry material (g), while mt defines as mass at the specific time (g).

2 $$\eta =\frac{\varDelta m}{\varDelta t}$$

Where ${\rm{\eta }}$ indicates dehydration rate (g/min), Δm defines as water loss between consecutive measurements (g), and Δt means time interval (min).

Mathematical modeling of drying curves was analyzed to fit the experimental data via Page’s and Logarithmic models according to previous study^36,42,43^.

### Water absorption expansion ratio and moisture Content

Fresh *T. fuciformis* soaked at 25 °C served as the control. Intact *T. fuciformis* (100 ± 0.2 g per sample) were blanched, cooled, drained, and weighed. The ratio of post-blanching to pre-blanching weight was defined as the water absorption expansion ratio^44^, with triplicate measurements per group.

Moisture content was determined using a moisture analyzer (HE53, Mettler Toledo Instrument (Shanghai) Co., Ltd.) based on the loss-on-drying principle at a drying temperature of 130 °C. Approximately 0.8 g of uniformly sliced sample was dried and measured in triplicate.

### Texture analysis

According to previous research with slight modifications^45,46^. The homogenized samples were assessed by a texture analyzer (TA.XT. PLUS, SMS, UK). following methods described by previous research with slight Modifications^47^: Each sample (3 g) was placed in a 24-well plate, ensuring even filling without voids. A P/5 probe was used with the settings (pre-test speed = 2 mm/s, test speed = 2 mm/s, post-test speed = 2 mm/s, compression ratio = 50%, trigger force = 5 g). Fresh homogenized *T. fuciformis* served as the control group (CG), with six replicates.

### Rehydration ratio

Based on a previous study with slight modifications^13,38^. Dried samples (1.00 ± 0.05 g) from different blanching conditions were rehydrated in 80 mL of water at 90 °C. Every 2 min, samples were drained through a 4 mm sieve, weighed, and recorded. Triplicate measurements were conducted at constant water temperature. The rehydration ratio was calculated using Eq. (3)

3 $$\mathrm{Rehydration}\,\mathrm{ratio}=\frac{{W}_{r}}{{W}_{d}}$$

Where *W*_r_ (g) defines as weight after rehydration, *W*_d_ (g) indicates weight of the dried sample.

### Water status and distribution of fresh *T. fuciformis* via pretreatment

Fresh *T. fuciformis* samples were used as the control group (CG). According to a previous study^17^, LF-NMR measurement: spin–spin relaxation time (T_2_) was measured by using a MesoMR23-060H-I NMR analyzer (Niumag Analytical Instruments Co., Suzhou, China) under constant temperature (32 °C), with a proton resonance frequency of 22.393 MHz. Intact fungal lobes weighing approximately 2 g were placed in a 60 mm diameter tube for later analysis. A CPMG Carr–Purcell–Meiboom–Gill sequence was employed with 90° and 180° pulse durations of 6.00 µs and 15.04 µs, respectively. Other parameters included a recycle delay of 6000 ms, receiver gain set to 2, 3 repeated scans per sample, an echo time of 0.8 ms, and 10,000 echoes. The resulting CPMG decay data were fitted using the SIRT algorithm with 100,000 iterations.

For MRI, approximately 5 g of fresh *T. fuciformis* samples with intact clustered structures were subjected to blanching, drained, and equilibrated to room temperature before imaging. T_2_-weighted images were acquired by using an NM42-060H-I NMR imager with a spin-echo (SE) sequence in the coronal plane. Repetition time (TR) and echo time (TE) were set to 300 ms and 20 ms, respectively, with 7 slices at a thickness of 10 mm per slice, and 4 scan averages. In T_2_-weighted images, regions with higher proton density exhibited stronger signals and appeared brighter.

### Color evaluation

Dried *T. fuciformis* was then crushed, wrapped in cling film, and compressed to prevent light transmission. A high-precision colorimeter (NR10QC, 3nh) measured surface color (L* = lightness, a* = redness, b* = yellowness), calibrated using a standard white tile (*L* = 94.12, *a* = −1.08, *b* = 2.12).

Chroma and total color difference (TCD and ΔE) were calculated via Eqs. (4) and (5), respectively^48^.

4 $$\mathrm{Chroma}=\sqrt{{a}^{2}+{b}^{2}}$$

5 $$\varDelta E=\sqrt{{(L-{L}_{0})}^{2}+{(a-{a}_{0})}^{2}+{(b-{b}_{0})}^{2}}$$

Where *L*_*0*_, *a*_*0*_, *b*_*0*_ represent the values of untreated CG.

### Water activity

The water activity of dried *T. fuciformis* was measured at room temperature by a water activity meter (AW-1, Shibata Scientific Technology Ltd.) while environmental stability was maintained (no direct measurement after refrigeration)^49^.

### Optical microscopy

Fresh and blanched (80 °C) *T. fuciformis* samples (4 × 4 mm) were flattened, mounted, and observed at 40 × magnification (Leica DM500). Images were captured via Capture2.4 software.

### SEM

According to previous reports with slight modifications^21^. Samples blanched at 80 °C for 5 min were fixed in paraformaldehyde (4 °C, 8 h), rinsed with phosphate buffer (0.1 mol/L, pH 7.2–7.4), dehydrated in ethanol, freeze-dried, sputter-coated with gold, and analyzed by Regulus 8100, Hitachi at 1k–30k× magnification (5 kV).

### Polysaccharide content

The phenol-sulfuric acid method was utilized to analyze the polysaccharide content of dried *T. fuciformis*^28^. The method was further modified according to the properties of *T. fuciformis* as follows: dried powder samples (1:100 w/v) were extracted in boiling water for 3 h, filtered, centrifuged, and diluted tenfold. Absorbance of the resulting solutions was measured (SpectraMax i3X, Molecular Devices) at 490 nm. D-glucose was used as the standard substance; the standard curve (Supplementary Fig. 4) equation was shown in Eq. (6):

6 $$y=6.0114x+0.0744\,\left({R}^{2}=0.9992\right)$$

The polysaccharide content was calculated in Eq. (7)

7 $$\omega =\frac{x\times 50\times 0.9}{m\times 0.2\div10}\times {10}^{-4}\times 100 \%$$

Where $y$ presents the absorbance per mL of sample solution, and the $x$ is the weight of polysaccharide per mL of sample solution (μg/mL), *ω* is the content of polysaccharide of the sample (%), *m* is the weight of the sample and 0.9 presents glucose correction factor.

### Rheology

Samples (1:40 w/v) were heated at 90 °C for 120 min, rapidly cooled, and homogenized for rheological testing (MCR302, Anton Paar). Rheological analysis was conducted based on modifications of the previous research^40,50^.

### Shear rheology

At 25 °C, apparent viscosity and shear stress (1–100 s⁻¹) were measured using a cone-plate (0.051 mm gap).

### Dynamic viscoelasticity

At 25 °C (1 Hz, 1% strain), storage modulus (*G’*) and loss modulus (*G”*) were measured (1–100 rad/s).

### Statistical analysis

The experiments were repeated in triplicate except for special instructions, and the results were expressed as the mean ± standard deviation (SD). Statistical analysis was performed using one-way analysis of variance (ANOVA) followed by Tukey’s multiple comparison post-hoc test to evaluate differences among treatment groups and the control at a 95% confidence level (*p* < 0.05). All statistical analyses were conducted using SPSS 16.0, and Origin 2018 was used for data visualization.

## Supplementary information

Supplementary information

## Data Availability

The datasets generated and analyzed during the current study are not publicly available due to laboratory data management regulations but are available from the corresponding author on reasonable request.
