# Supplementary material for: Beyond conventional drying: improving the quality of dried Tremella fuciformis via a heat-integrated pretreatment
Source: NPJ Sci Food. 2026 Mar 23;10:154. doi: 10.1038/s41538-026-00805-8 (PMC13171875; doi:10.1038/s41538-026-00805-8)
Supplement: Supplementary file 1 — Supplementary information [file 41538_2026_805_MOESM1_ESM.doc]

**Supplementary Information**

Beyond Conventional Drying: Improving the Quality of Dried *Tremella fuciformis* via a Heat-Integrated Pretreatment

Yuanhui ZHANG, Nengpai SHI, Cong YANG, Yuankun JIA, Jiaxuan PENG, Xuemei HOU, Shengnan LIN *, Xiangyang LIN *

*College of Biological Science and Engineering, Fuzhou University, Fuzhou, Fujian 350108, PR China.*

Yuanhui ZHANG: 240827002@fzu.edu.cn

Nengpai SHI: 240827040@ fzu.edu.cn

Cong YANG: 082402130@fzu.edu.cn

Yuankun JIA: 082403209@fzu.edu.cn

Jiaxuan PENG: 082402122@fzu.edu.cn

Xuemei HOU: xuemeihou@fzu.edu.cn

**Corresponding Author**

* Shengnan LIN, College of Biological Science and Engineering, Fuzhou University, Fuzhou 350108, People's Republic of China. E-mail: shengnan.lin@fzu.edu.cn

** Xiangyang LIN, College of Biological Science and Engineering, Fuzhou University, Fuzhou 350108, People's Republic of China. E-mail: xylin@fzu.edu.cn


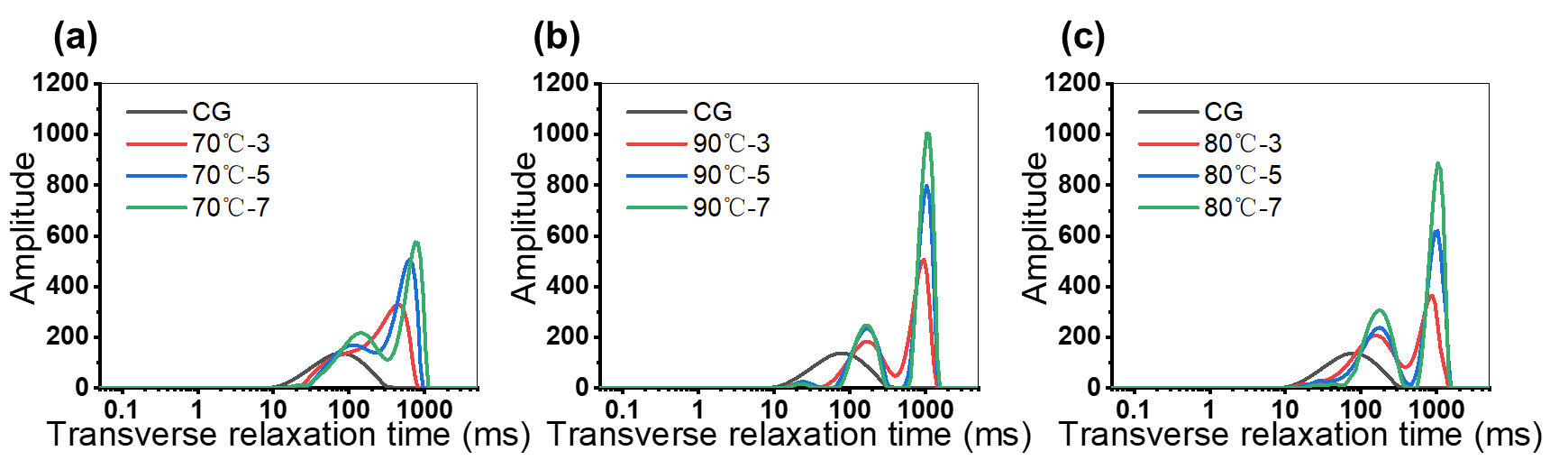


**Supplementary Figure. 1|** Effects of different heat-integrated pretreatments on the transverse relaxation time (T_2_): (a) 70℃, (b) 80℃, (c) 90℃.


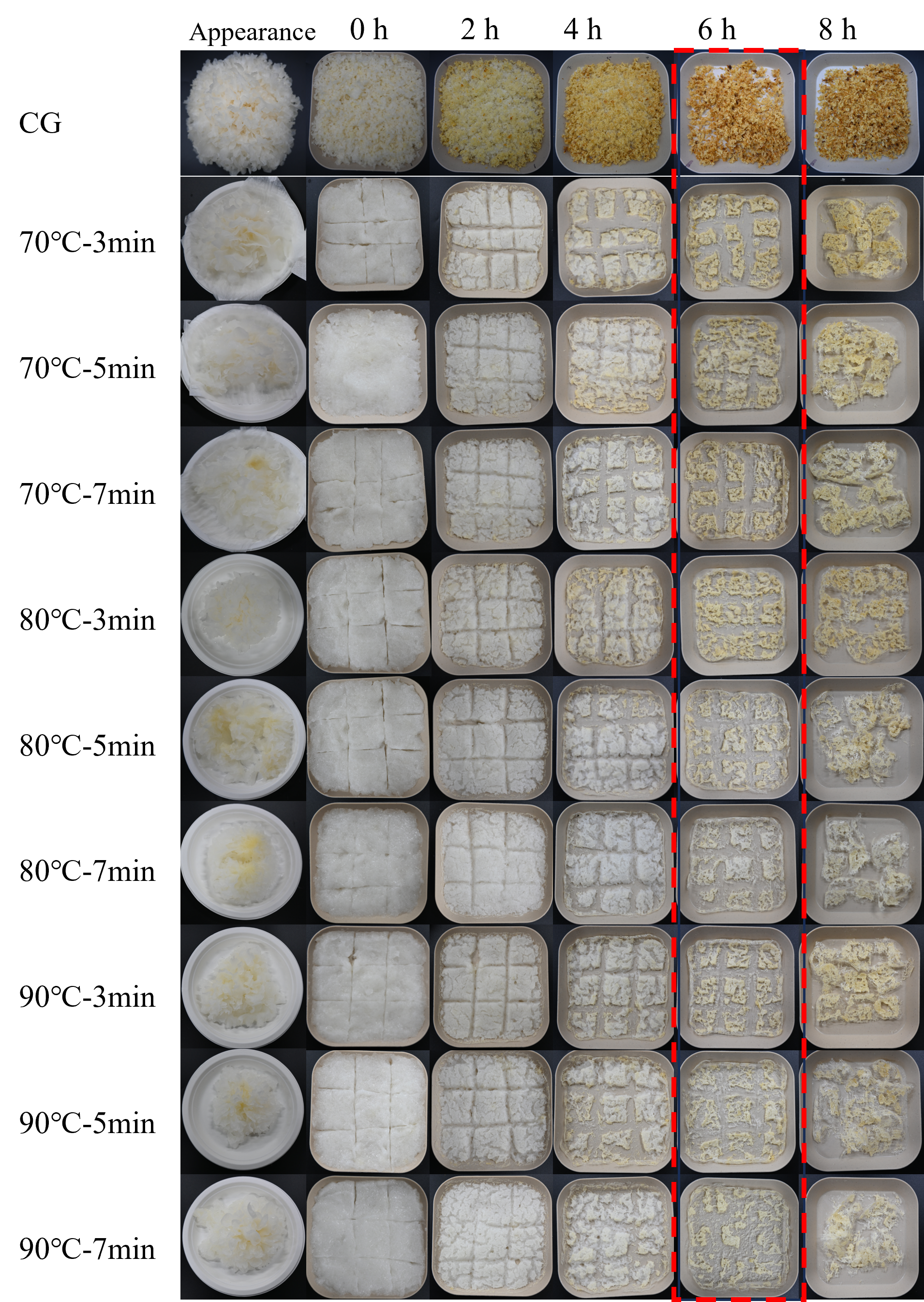


**Supplementary Figure. 2|** Appearance and drying processing of *T. fuciformis*, photographed at 2 h intervals.


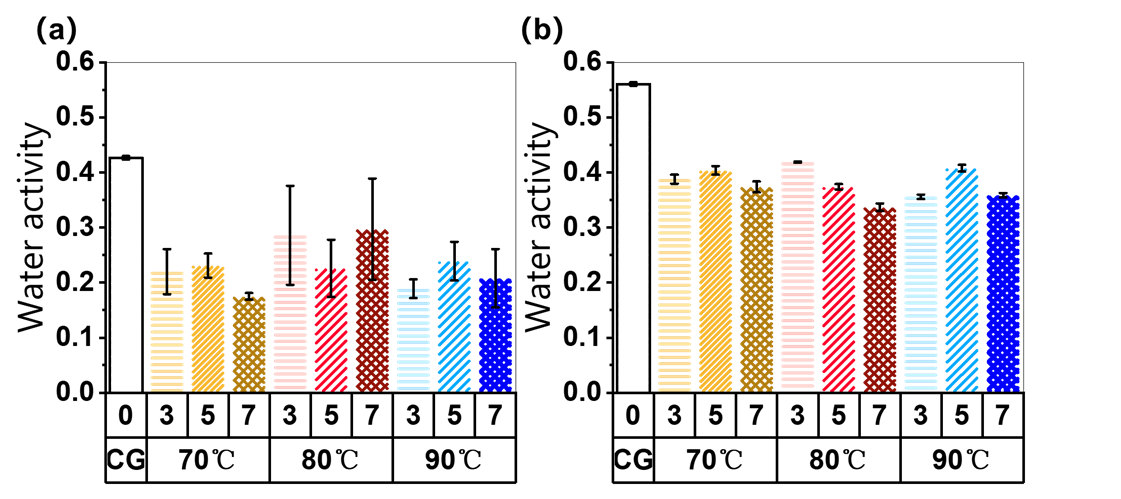


**Supplementary Figure. 3|** Water activity of the dried *T. fuciformis* samples during sealed refrigerated storage after (a) two weeks and (b) eight weeks.

**Supplementary Figure. 4|** Calibration curve for total sugar determination

**Supplementary Table. 1|** Relative moisture contents and relaxation time of fresh *T. fuciformis* via different heat-integrated pretreatments.

| **Groups** | | **M_2_** | **M_21_(%)** | **M_22_(%)** | **M_23_(%)** | **T_21_(ms)** | **T_22_(ms)** | **T_23_(ms)** |
| --- | --- | --- | --- | --- | --- | --- | --- | --- |
| **Temperature** | **Time** |  |  |  |  |  |  |  |
| **CG** | | 3380.11±57.50 | 2.12±0.79 | 97.31±0.92 | 0.56±0.16 | 1.16±0.36； 9.72±3.28 | 79.68±23.82 | 453.10±159.11 |
| **70℃** | **3 min** | 7183.29±328.33 b | 0.15±0.22 b | 99.90±0.17 | | 9.20±5.44 | 449.66±43.12 | |
|  | **5 min** | 8400.24±414.43 a | 0.44±0.25 a | 37.37±3.83 | 62.19±4.00 | 16.91±5.96 | 113.71±13.77 | 636.83±0.00 |
|  | **7 min** | 9061.92±252.82 a | 0.78±0.27 a | 45.07±2.04 | 54.15±2.28 | 23.05±1.87 | 140.52±6.66 | 790.93±36.47 |
| **80℃** | **3 min** | 7312.02±358.84 c | 1.39±0.46 | 55.89±1.86 a | 43.19±1.14 b | 18.04±0.00 | 156.89±15.05 | 888.21±143.41 |
|  | **5 min** | 8530.81±435.40 b | 2.74±1.63 | 42.53±1.43 b | 54.73±0.87 a | 27.93±3.51 | 180.69±29.24 | 1041.94±139.82 |
|  | **7 min** | 9851.32±620.75 a | 2.52±2.42 | 39.55±5.74 b | 58.77±6.92 a | 35.51±11.97 | 174.64±14.13 | 1064.44±50.43 |
| **90℃** | **3 min** | 6977.73±386.21 c | 2.04±0.47 ab | 40.58±0.79 a | 57.38±0.34 c | 21.26±1.72 | 174.263±0.00 | 956.86±77.44 |
|  | **5 min** | 8189.11±283.59 b | 2.36±0.31 b | 33.63±1.09 b | 64.01±1.10 b | 24.30±1.12 | 169.74±7.83 | 1066.70±96.93 |
|  | **7 min** | 9051.58±287.07 a | 1.30±0.41 a | 28.41±2.88 c | 70.29±3.28 a | 21.91±2.76 | 169.74±7.83 | 1093.55±50.43 |
| **Temperature** | | * | * | ** | ** | ** | ** | * |
| **Time** | | ** |  | ** | ** | ** |  | ** |
| **Temperature*time** | |  |  | * | * |  |  |  |

Note: *p* ≤ 0.05 is considered significant (*) and p ≤ 0.01 highly significant (**). Within groups, different letters in the same column indicate significant differences (p ≤ 0.05).
